# 1-[Bicyclo[4.2.0]octa-1(6),2,4-trien-3-yl]-3-[bicyclo[4.2.0]octa-1(6),2,4-trien-3-yl­methyl]imidazolium hexa­fluoro­phos­phate

**DOI:** 10.1107/S1600536807067086

**Published:** 2008-01-11

**Authors:** Fang-Hua Zhu, Jun-Xiao Yang, Zhi-Hua Mao, Ru-Gang Xie

**Affiliations:** a College of Chemistry, Sichuan University, Chengdu 610064, People’s Republic of China; b School of Materials Science and Engineering, Southwest University of Science and Technology, Mianyang 621010, People’s Republic of China; c Analytical and Testing Center, Sichuan University, Chengdu 610064, People’s Republic of China

## Abstract

In the title compound, C_20_H_19_N_2_^+^·PF_6_^−^, the two benzocyclo­butene units are essentially planar and they form dihedral angles of 38.0 (2) and 72.7 (2)°, with the central imidazolium ring. In the crystal structure, weak C—H⋯π and π-–π stacking inter­actions [centroid–centroid distance = 3.742 (2) Å] contribute to the stability of the crystal structure. The PF_6_^−^ ion is disordered over two positions with site occupancies of 0.869 (9) and 0.131 (9).

## Related literature

For related literature, see: Farona (1996 ▶); Kirchhoff & Bruza (1993 ▶); Michellys *et al.* (2001 ▶); Nemeto & Fukumoto (1998 ▶); Zhang *et al.* (2006 ▶).
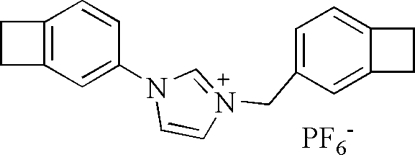


## Experimental

### 

#### Crystal data

C_20_H_19_N_2_^+^·F_6_P^−^
*M* *_r_* = 432.34
Triclinic, 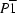

*a* = 9.311 (3) Å
*b* = 10.138 (3) Å
*c* = 10.562 (3) Å
α = 86.82 (2)°
β = 86.44 (2)°
γ = 73.61 (2)°
*V* = 953.9 (5) Å^3^
*Z* = 2
Mo *K*α radiation
μ = 0.21 mm^−1^
*T* = 296 (2) K
0.28 × 0.25 × 0.18 mm

#### Data collection

Enraf–Nonius CAD-4 diffractometer
Absorption correction: none
3558 measured reflections
3522 independent reflections
2238 reflections with *I* > 2σ(*I*)
*R*_int_ = 0.006
3 standard reflections every 300 reflections intensity decay: 4.6%

#### Refinement

*R*[*F* ^2^ > 2σ(*F* ^2^)] = 0.075
*wR*(*F* ^2^) = 0.223
*S* = 1.15
3522 reflections
299 parameters
81 restraints
H-atom parameters constrained
Δρ_max_ = 0.64 e Å^−3^
Δρ_min_ = −0.41 e Å^−3^

### 

Data collection: *DIFRAC* (Gabe & White, 1993 ▶); cell refinement: *DIFRAC*; data reduction: *NRCVAX* (Gabe *et al.*, 1989 ▶); program(s) used to solve structure: *SHELXS97* (Sheldrick, 1997 ▶); program(s) used to refine structure: *SHELXL97* (Sheldrick, 1997 ▶); molecular graphics: *ORTEPIII* (Burnett & Johnson, 1996 ▶); software used to prepare material for publication: *SHELXL97* and *PLATON* (Spek, 2003 ▶).

## Supplementary Material

Crystal structure: contains datablocks global, I. DOI: 10.1107/S1600536807067086/ci2536sup1.cif

Structure factors: contains datablocks I. DOI: 10.1107/S1600536807067086/ci2536Isup2.hkl

Additional supplementary materials:  crystallographic information; 3D view; checkCIF report

## Figures and Tables

**Table 1:** Hydrogen-bond geometry (Å, °) *Cg*1 is the centroid of the C3–C8 ring.

*D*—H⋯*A*	*D*—H	H⋯*A*	*D*⋯*A*	*D*—H⋯*A*
C2—H2*B*⋯*Cg*1^i^	0.97	3.00	3.813 (5)	143

Symmetry code: (i) 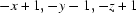.

**Fig. 1.**
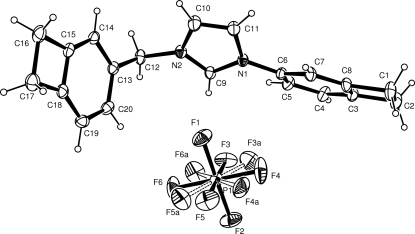
The molecular structure of the title compound, showing 30% probability displacement ellipsoids and the atomic numbering.

## supplementary crystallographic information

### Comment

Benzocyclobutenes (BCBs) are important building blocks for new polymers and advanced materials (Nemeto & Fukumoto, 1998; Michellys *et al.*, 2001). Most of the reported benzocyclobutene monomers in the literature are bis-BCB-functionalized compounds owing to their highly crosslinked structure and their excellent properties such as the insolubility, low solvent pickup and good thermal stability (Farona, 1996; Zhang *et al.*, 2006; Kirchhoff & Bruza, 1993). We report here the crystal structure of the title compound, a novel bisbenzocyclobutene-terminated imidazolium which was obtained by an anion metathesis between 1-(4-benzocyclobutenyl)-3-(4-benzocyclobutenylmethyl) imidazolium choloride and hexafluorophosphate ammonium.

The two benzocyclobutene units are essentially planar. The plane of the C1—C8 and C13—C20 benzocyclobutene units form dihedral angles of 38.0 (2) and 72.7 (2)°, respectively, with the central imidazolium ring.

A combination of intermolecular π-π and C—H···π interactions provide packing forces in the crystal structure of the title compound. A π-π interaction between C13—C15/C18—C20 benzene ring and its symmetry- related counterpart at (-*x*, 1 - *y*, -*z*), with their centroids separated by 3.742 (2) Å, plays an important part in the connection of two adjacent molecules. In addition, a weak C—H···π interaction between C2—H2B group and C3—C8 benzene ring at (1 - *x*, -1 - *y*, 1 - *z*) contributes to the crystal packing (Table 1).

### Experimental

4-(*N*-imidazolyl)benzocyclobutene (5 mmol, 850 mg) and 4-cholomethylbenzocyclobutene (5 mmol, 760 mg) were placed in a two-necked round-bottomed flask under a nitrogen atmosphere, and the mixture was heated at 353 K for 3 h. After the reaction was completed, the resulting 1-(4-benzocyclobutenyl)-3-(4-benzocyclobutenylmethyl)imidazolium chloride, [BBMI][Cl^-^], was obtained in 85% yield (1.381 g). [BBMI][Cl^-^] was dissolved in deioned water, and NH_4_PF_6_ was added to replace Cl^-^ ions by PF_6_^-^ ions, to obtain the title compound as a white solid (yield 1.331 g). Colourless crystals of the title compound were obtained by recrystallization of the solid from acetone and ethanol (1:1 *v*/*v*). ^1^ H NMR (400 MHz, CDCl_3_,): δ 8.84 (s, 1H), 7.41 (t, 1H), 7.31 (m, 2H), 7.27 (2 d, 1H), 7.22 (d, 1H), 7.18 (d, J = 8.0 Hz, 1H), 7.15 (s, 1H), 7.09 (d, J = 7.6 Hz, 1H), 5.37 (s, 2H), 3.21 (s, 4H), 3.17 (s, 4H); ^13^C NMR (100 MHz, CDCl_3_,): δ 148.58, 148.04, 7.65, 147.21, 133.71, 133.42, 130.78, 128.20, 124.45, 123.53, 122.77, 121.88, 121.46, 117.09, 54.52, 29.47, 29.32 p.p.m.

### Refinement

H atoms were positioned geometrically and refined in the riding model approximation with C—H = 0.93 or 0.97 Å and *U*_iso_(H) = 1.2*U*_eq_(C). Four F atoms of the PF_6_^-^ ion are disordered over two positions (F3,F4,F5,F6/F3A,F4A,F5A,F6A) with refined occupancies of 0.869 (9) and 0.131 (9). The disordered F atoms were restrained to be coplanar, with the F···F distances restrained to be equal. The displacement parameters of disordered F atoms were restrained to be approximately isotropic.

### Crystal data

**Table:**

C_20_H_19_N_2_^+^·F_6_P^–^	*Z* = 2
*M**_r_* = 432.34	*F*_000_ = 444
Triclinic, *P*1	*D*_x_ = 1.505 Mg m^−^^3^
Hall symbol: -P 1	Mo *K*α radiation λ = 0.71073 Å
*a* = 9.311 (3) Å	Cell parameters from 20 reflections
*b* = 10.138 (3) Å	θ = 4.9–9.1º
*c* = 10.562 (3) Å	µ = 0.21 mm^−^^1^
α = 86.82 (2)º	*T* = 296 (2) K
β = 86.44 (2)º	Block, colourless
γ = 73.61 (2)º	0.28 × 0.25 × 0.18 mm
*V* = 953.9 (5) Å^3^	

### Data collection

**Table:**

Enraf–Nonius CAD-4 diffractometer	*R*_int_ = 0.006
Radiation source: fine-focus sealed tube	θ_max_ = 25.5º
Monochromator: graphite	θ_min_ = 1.9º
*T* = 296(2) K	*h* = −10→11
ω/2θ scans	*k* = −2→12
Absorption correction: none	*l* = −12→12
3558 measured reflections	3 standard reflections
3522 independent reflections	every 300 reflections
2238 reflections with *I* > 2σ(*I*)	intensity decay: 4.6%

### Refinement

**Table:**

Refinement on *F*^2^	Secondary atom site location: difference Fourier map
Least-squares matrix: full	Hydrogen site location: inferred from neighbouring sites
*R*[*F*^2^ > 2σ(*F*^2^)] = 0.075	H-atom parameters constrained
*wR*(*F*^2^) = 0.223	*w* = 1/[σ^2^(*F*_o_^2^) + (0.1349*P*)^2^] where *P* = (*F*_o_^2^ + 2*F*_c_^2^)/3
*S* = 1.15	(Δ/σ)_max_ = 0.001
3522 reflections	Δρ_max_ = 0.64 e Å^−^^3^
299 parameters	Δρ_min_ = −0.41 e Å^−^^3^
81 restraints	Extinction correction: none
Primary atom site location: structure-invariant direct methods	

### Special details

**Table:**

Geometry. All e.s.d.'s (except the e.s.d. in the dihedral angle between two l.s. planes) are estimated using the full covariance matrix. The cell e.s.d.'s are taken into account individually in the estimation of e.s.d.'s in distances, angles and torsion angles; correlations between e.s.d.'s in cell parameters are only used when they are defined by crystal symmetry. An approximate (isotropic) treatment of cell e.s.d.'s is used for estimating e.s.d.'s involving l.s. planes.
Refinement. Refinement of *F*^2^ against ALL reflections. The weighted *R*-factor *wR* and goodness of fit *S* are based on *F*^2^, conventional *R*-factors *R* are based on *F*, with *F* set to zero for negative *F*^2^. The threshold expression of *F*^2^ > σ(*F*^2^) is used only for calculating *R*-factors(gt) *etc*. and is not relevant to the choice of reflections for refinement. *R*-factors based on *F*^2^ are statistically about twice as large as those based on *F*, and *R*- factors based on ALL data will be even larger.

### Fractional atomic coordinates and isotropic or equivalent isotropic displacement parameters (Å^2^)

**Table:**

	*x*	*y*	*z*	*U*_iso_*/*U*_eq_	Occ. (<1)
N1	0.3258 (3)	−0.0608 (3)	0.3056 (3)	0.0388 (7)	
N2	0.1779 (3)	0.1062 (3)	0.1997 (3)	0.0412 (7)	
C1	0.7078 (6)	−0.4108 (5)	0.5742 (4)	0.0654 (13)	
H1A	0.6556	−0.4310	0.6520	0.078*	
H1B	0.7911	−0.3758	0.5917	0.078*	
C2	0.7481 (5)	−0.5286 (5)	0.4784 (4)	0.0658 (13)	
H2A	0.8522	−0.5547	0.4476	0.079*	
H2B	0.7152	−0.6086	0.5066	0.079*	
C3	0.6426 (4)	−0.4283 (4)	0.3899 (4)	0.0486 (10)	
C4	0.5802 (5)	−0.4138 (4)	0.2742 (4)	0.0534 (11)	
H4	0.6062	−0.4838	0.2166	0.064*	
C5	0.4756 (4)	−0.2881 (4)	0.2472 (3)	0.0477 (10)	
H5	0.4297	−0.2731	0.1700	0.057*	
C6	0.4397 (4)	−0.1854 (4)	0.3348 (3)	0.0385 (8)	
C7	0.5044 (4)	−0.2001 (4)	0.4520 (3)	0.0452 (9)	
H7	0.4809	−0.1304	0.5099	0.054*	
C8	0.6057 (5)	−0.3256 (4)	0.4753 (3)	0.0474 (9)	
C9	0.2990 (4)	0.0017 (3)	0.1915 (3)	0.0381 (8)	
H9	0.3572	−0.0247	0.1175	0.046*	
C10	0.1233 (5)	0.1122 (4)	0.3233 (4)	0.0583 (12)	
H10	0.0378	0.1759	0.3552	0.070*	
C11	0.2151 (5)	0.0095 (4)	0.3902 (4)	0.0525 (10)	
H11	0.2059	−0.0104	0.4768	0.063*	
C12	0.1133 (4)	0.2028 (4)	0.0937 (3)	0.0457 (9)	
H12A	0.0101	0.2026	0.0854	0.055*	
H12B	0.1688	0.1715	0.0150	0.055*	
C13	0.1181 (4)	0.3462 (4)	0.1145 (3)	0.0394 (8)	
C14	−0.0027 (4)	0.4358 (4)	0.1797 (3)	0.0414 (9)	
H14	−0.0882	0.4103	0.2072	0.050*	
C15	0.0126 (4)	0.5639 (4)	0.2006 (3)	0.0446 (9)	
C16	−0.0632 (5)	0.7005 (4)	0.2603 (5)	0.0611 (12)	
H16A	−0.0769	0.6945	0.3520	0.073*	
H16B	−0.1550	0.7522	0.2215	0.073*	
C17	0.0771 (5)	0.7460 (4)	0.2122 (4)	0.0613 (12)	
H17A	0.0569	0.8218	0.1496	0.074*	
H17B	0.1360	0.7622	0.2793	0.074*	
C18	0.1361 (4)	0.6058 (4)	0.1564 (3)	0.0448 (9)	
C19	0.2544 (4)	0.5185 (4)	0.0891 (4)	0.0494 (10)	
H19	0.3375	0.5464	0.0583	0.059*	
C20	0.2429 (4)	0.3886 (4)	0.0702 (3)	0.0465 (10)	
H20	0.3209	0.3271	0.0264	0.056*	
P1	0.67915 (11)	0.11989 (10)	0.17994 (9)	0.0466 (4)	
F1	0.5204 (3)	0.1791 (4)	0.2485 (3)	0.0890 (10)	
F2	0.8367 (3)	0.0567 (3)	0.1101 (3)	0.0964 (11)	
F3	0.6267 (4)	0.0006 (4)	0.1205 (3)	0.0908 (17)	0.869 (9)
F4	0.7321 (5)	0.0220 (4)	0.2990 (3)	0.109 (2)	0.869 (9)
F5	0.7340 (4)	0.2334 (4)	0.2388 (5)	0.112 (2)	0.869 (9)
F6	0.6275 (5)	0.2124 (5)	0.0586 (4)	0.120 (2)	0.869 (9)
F3A	0.6793 (7)	−0.018 (2)	0.2185 (12)	0.127 (14)	0.131 (9)
F4A	0.7527 (10)	0.1314 (12)	0.3022 (15)	0.090 (10)	0.131 (9)
F5A	0.6805 (7)	0.260 (2)	0.1369 (12)	0.103 (11)	0.131 (9)
F6A	0.6104 (10)	0.1040 (12)	0.0613 (16)	0.093 (10)	0.131 (9)

### Atomic displacement parameters (Å^2^)

**Table:**

	*U*^11^	*U*^22^	*U*^33^	*U*^12^	*U*^13^	*U*^23^
N1	0.0443 (17)	0.0364 (15)	0.0320 (15)	−0.0048 (13)	−0.0036 (12)	−0.0025 (12)
N2	0.0480 (18)	0.0395 (16)	0.0332 (15)	−0.0068 (14)	−0.0038 (13)	−0.0028 (12)
C1	0.075 (3)	0.066 (3)	0.047 (2)	−0.005 (2)	−0.023 (2)	0.011 (2)
C2	0.064 (3)	0.065 (3)	0.056 (3)	0.002 (2)	−0.013 (2)	0.012 (2)
C3	0.046 (2)	0.051 (2)	0.042 (2)	−0.0017 (18)	−0.0069 (16)	0.0010 (17)
C4	0.050 (2)	0.055 (2)	0.046 (2)	0.0046 (18)	−0.0117 (17)	−0.0145 (18)
C5	0.049 (2)	0.055 (2)	0.0348 (19)	−0.0043 (18)	−0.0110 (16)	−0.0087 (17)
C6	0.0361 (18)	0.0414 (19)	0.0340 (17)	−0.0041 (15)	−0.0050 (14)	0.0004 (14)
C7	0.057 (2)	0.048 (2)	0.0287 (17)	−0.0099 (18)	−0.0066 (16)	−0.0024 (15)
C8	0.055 (2)	0.050 (2)	0.0331 (18)	−0.0094 (18)	−0.0107 (16)	0.0070 (16)
C9	0.044 (2)	0.0407 (19)	0.0286 (16)	−0.0088 (16)	−0.0053 (14)	−0.0017 (14)
C10	0.068 (3)	0.049 (2)	0.042 (2)	0.008 (2)	0.0080 (19)	−0.0024 (18)
C11	0.061 (3)	0.050 (2)	0.0356 (19)	0.0015 (19)	0.0081 (18)	−0.0034 (16)
C12	0.052 (2)	0.046 (2)	0.0351 (18)	−0.0040 (17)	−0.0135 (16)	−0.0021 (16)
C13	0.0406 (19)	0.0434 (19)	0.0305 (17)	−0.0041 (15)	−0.0117 (14)	0.0019 (14)
C14	0.0363 (19)	0.043 (2)	0.0417 (19)	−0.0050 (15)	−0.0094 (15)	0.0040 (15)
C15	0.041 (2)	0.043 (2)	0.044 (2)	−0.0004 (16)	−0.0123 (16)	0.0019 (16)
C16	0.058 (3)	0.043 (2)	0.075 (3)	−0.0011 (19)	−0.008 (2)	−0.008 (2)
C17	0.063 (3)	0.050 (2)	0.071 (3)	−0.015 (2)	−0.014 (2)	−0.002 (2)
C18	0.045 (2)	0.047 (2)	0.0414 (19)	−0.0100 (17)	−0.0131 (16)	0.0077 (16)
C19	0.042 (2)	0.064 (3)	0.044 (2)	−0.0179 (19)	−0.0051 (17)	0.0037 (18)
C20	0.040 (2)	0.056 (2)	0.0373 (19)	−0.0024 (17)	−0.0069 (16)	−0.0042 (17)
P1	0.0469 (6)	0.0481 (6)	0.0409 (6)	−0.0061 (5)	−0.0024 (4)	−0.0045 (4)
F1	0.0595 (17)	0.125 (2)	0.0650 (17)	0.0038 (16)	0.0102 (13)	−0.0246 (17)
F2	0.0592 (18)	0.098 (2)	0.127 (3)	−0.0126 (16)	0.0273 (18)	−0.042 (2)
F3	0.093 (3)	0.122 (4)	0.078 (3)	−0.060 (3)	0.027 (2)	−0.048 (3)
F4	0.165 (4)	0.074 (3)	0.054 (2)	0.023 (3)	−0.021 (2)	0.0058 (18)
F5	0.105 (3)	0.082 (3)	0.158 (5)	−0.031 (2)	0.003 (3)	−0.061 (3)
F6	0.151 (4)	0.106 (4)	0.060 (2)	0.024 (3)	0.002 (2)	0.034 (2)
F3A	0.122 (19)	0.094 (16)	0.18 (2)	−0.059 (14)	−0.017 (16)	0.033 (16)
F4A	0.077 (13)	0.105 (19)	0.079 (13)	−0.006 (12)	−0.049 (11)	0.013 (12)
F5A	0.140 (19)	0.086 (15)	0.091 (17)	−0.050 (13)	0.001 (14)	0.004 (13)
F6A	0.145 (18)	0.095 (17)	0.052 (12)	−0.048 (14)	−0.024 (11)	−0.012 (12)

### Geometric parameters (Å, °)

**Table:**

N1—C9	1.334 (4)	C12—H12B	0.97
N1—C11	1.382 (4)	C13—C20	1.395 (5)
N1—C6	1.435 (4)	C13—C14	1.400 (5)
N2—C9	1.313 (4)	C14—C15	1.378 (5)
N2—C10	1.370 (5)	C14—H14	0.93
N2—C12	1.484 (4)	C15—C18	1.381 (6)
C1—C8	1.517 (5)	C15—C16	1.518 (5)
C1—C2	1.558 (6)	C16—C17	1.552 (7)
C1—H1A	0.97	C16—H16A	0.97
C1—H1B	0.97	C16—H16B	0.97
C2—C3	1.522 (5)	C17—C18	1.510 (5)
C2—H2A	0.97	C17—H17A	0.97
C2—H2B	0.97	C17—H17B	0.97
C3—C4	1.368 (5)	C18—C19	1.387 (5)
C3—C8	1.371 (5)	C19—C20	1.378 (5)
C4—C5	1.395 (5)	C19—H19	0.93
C4—H4	0.93	C20—H20	0.93
C5—C6	1.386 (5)	P1—F3A	1.43 (3)
C5—H5	0.93	P1—F5A	1.47 (2)
C6—C7	1.393 (5)	P1—F6A	1.48 (2)
C7—C8	1.373 (5)	P1—F4A	1.524 (18)
C7—H7	0.93	P1—F5	1.556 (4)
C9—H9	0.93	P1—F6	1.563 (4)
C10—C11	1.343 (5)	P1—F4	1.572 (4)
C10—H10	0.93	P1—F1	1.576 (3)
C11—H11	0.93	P1—F2	1.579 (3)
C12—C13	1.497 (5)	P1—F3	1.597 (4)
C12—H12A	0.97		
			
C9—N1—C11	107.5 (3)	C15—C14—H14	122.0
C9—N1—C6	126.8 (3)	C13—C14—H14	122.0
C11—N1—C6	125.4 (3)	C14—C15—C18	123.0 (3)
C9—N2—C10	108.2 (3)	C14—C15—C16	144.1 (4)
C9—N2—C12	125.8 (3)	C18—C15—C16	93.0 (3)
C10—N2—C12	125.9 (3)	C15—C16—C17	86.7 (3)
C8—C1—C2	86.7 (3)	C15—C16—H16A	114.2
C8—C1—H1A	114.2	C17—C16—H16A	114.2
C2—C1—H1A	114.2	C15—C16—H16B	114.2
C8—C1—H1B	114.2	C17—C16—H16B	114.2
C2—C1—H1B	114.2	H16A—C16—H16B	111.4
H1A—C1—H1B	111.4	C18—C17—C16	86.8 (3)
C3—C2—C1	86.3 (3)	C18—C17—H17A	114.2
C3—C2—H2A	114.3	C16—C17—H17A	114.2
C1—C2—H2A	114.3	C18—C17—H17B	114.2
C3—C2—H2B	114.3	C16—C17—H17B	114.2
C1—C2—H2B	114.3	H17A—C17—H17B	111.3
H2A—C2—H2B	111.4	C15—C18—C19	121.1 (4)
C4—C3—C8	122.4 (3)	C15—C18—C17	93.5 (3)
C4—C3—C2	144.0 (4)	C19—C18—C17	145.3 (4)
C8—C3—C2	93.5 (3)	C20—C19—C18	116.7 (4)
C3—C4—C5	116.3 (3)	C20—C19—H19	121.6
C3—C4—H4	121.8	C18—C19—H19	121.6
C5—C4—H4	121.8	C19—C20—C13	122.2 (3)
C6—C5—C4	120.5 (3)	C19—C20—H20	118.9
C6—C5—H5	119.8	C13—C20—H20	118.9
C4—C5—H5	119.8	F3A—P1—F5A	178.4 (5)
C5—C6—C7	123.0 (3)	F3A—P1—F6A	88.8 (6)
C5—C6—N1	118.5 (3)	F5A—P1—F6A	90.0 (6)
C7—C6—N1	118.5 (3)	F3A—P1—F4A	89.6 (6)
C8—C7—C6	114.7 (3)	F5A—P1—F4A	91.5 (6)
C8—C7—H7	122.7	F6A—P1—F4A	178.2 (6)
C6—C7—H7	122.7	F5—P1—F6	92.2 (3)
C3—C8—C7	123.1 (3)	F5—P1—F4	89.7 (3)
C3—C8—C1	93.5 (3)	F6—P1—F4	177.9 (2)
C7—C8—C1	143.4 (3)	F3A—P1—F1	91.1 (2)
N2—C9—N1	109.6 (3)	F5A—P1—F1	90.1 (2)
N2—C9—H9	125.2	F6A—P1—F1	91.5 (2)
N1—C9—H9	125.2	F4A—P1—F1	89.5 (2)
C11—C10—N2	107.8 (3)	F5—P1—F1	89.43 (19)
C11—C10—H10	126.1	F6—P1—F1	90.82 (19)
N2—C10—H10	126.1	F4—P1—F1	90.17 (19)
C10—C11—N1	106.8 (3)	F3A—P1—F2	87.5 (2)
C10—C11—H11	126.6	F5A—P1—F2	91.3 (2)
N1—C11—H11	126.6	F6A—P1—F2	87.4 (2)
N2—C12—C13	111.8 (3)	F4A—P1—F2	91.5 (2)
N2—C12—H12A	109.3	F5—P1—F2	92.30 (19)
C13—C12—H12A	109.3	F6—P1—F2	89.4 (2)
N2—C12—H12B	109.3	F4—P1—F2	89.5 (2)
C13—C12—H12B	109.3	F1—P1—F2	178.25 (18)
H12A—C12—H12B	107.9	F5—P1—F3	178.5 (2)
C20—C13—C14	120.9 (3)	F6—P1—F3	88.8 (3)
C20—C13—C12	119.8 (3)	F4—P1—F3	89.3 (2)
C14—C13—C12	119.3 (3)	F1—P1—F3	91.61 (18)
C15—C14—C13	116.0 (4)	F2—P1—F3	86.66 (17)
			
C8—C1—C2—C3	0.8 (3)	C12—N2—C10—C11	−178.3 (4)
C1—C2—C3—C4	−177.7 (7)	N2—C10—C11—N1	−0.8 (5)
C1—C2—C3—C8	−0.9 (4)	C9—N1—C11—C10	0.7 (5)
C8—C3—C4—C5	−0.3 (7)	C6—N1—C11—C10	−174.1 (4)
C2—C3—C4—C5	176.0 (6)	C9—N2—C12—C13	−115.5 (4)
C3—C4—C5—C6	0.3 (7)	C10—N2—C12—C13	63.2 (5)
C4—C5—C6—C7	0.4 (7)	N2—C12—C13—C20	89.8 (4)
C4—C5—C6—N1	−176.8 (4)	N2—C12—C13—C14	−89.5 (4)
C9—N1—C6—C5	−35.6 (6)	C20—C13—C14—C15	−2.4 (5)
C11—N1—C6—C5	138.2 (4)	C12—C13—C14—C15	177.0 (3)
C9—N1—C6—C7	147.2 (4)	C13—C14—C15—C18	2.5 (5)
C11—N1—C6—C7	−39.0 (5)	C13—C14—C15—C16	−178.8 (5)
C5—C6—C7—C8	−0.9 (6)	C14—C15—C16—C17	−179.7 (5)
N1—C6—C7—C8	176.2 (3)	C18—C15—C16—C17	−0.8 (3)
C4—C3—C8—C7	−0.3 (7)	C15—C16—C17—C18	0.8 (3)
C2—C3—C8—C7	−178.1 (4)	C14—C15—C18—C19	−1.0 (6)
C4—C3—C8—C1	178.7 (4)	C16—C15—C18—C19	179.7 (3)
C2—C3—C8—C1	0.9 (4)	C14—C15—C18—C17	−179.9 (3)
C6—C7—C8—C3	0.9 (6)	C16—C15—C18—C17	0.9 (3)
C6—C7—C8—C1	−177.5 (6)	C16—C17—C18—C15	−0.9 (3)
C2—C1—C8—C3	−0.9 (4)	C16—C17—C18—C19	−179.2 (6)
C2—C1—C8—C7	177.7 (6)	C15—C18—C19—C20	−0.7 (5)
C10—N2—C9—N1	−0.2 (4)	C17—C18—C19—C20	177.3 (5)
C12—N2—C9—N1	178.7 (3)	C18—C19—C20—C13	0.8 (5)
C11—N1—C9—N2	−0.3 (4)	C14—C13—C20—C19	0.8 (5)
C6—N1—C9—N2	174.4 (3)	C12—C13—C20—C19	−178.6 (3)
C9—N2—C10—C11	0.6 (5)		

### Hydrogen-bond geometry (Å, °)

**Table:**

*D*—H···*A*	*D*—H	H···*A*	*D*···*A*	*D*—H···*A*
C2—H2B···Cg1^i^	0.97	3.00	3.813 (5)	143

Symmetry codes: (i) −*x*+1, −*y*−1, −*z*+1.
